# The Relations of Residents to Students

**Published:** 1907-06-08

**Authors:** 


### Resident Medical Officers' Department.

[Contributions to this Column are invited, and, if accepted, will be paid for.]

### THE RELATIONS OF RESIDENTS TO STUDENTS.

The subject of the relation of hospital residents to students is one of much interest, for it has a considerable bearing upon the wider question of medical education. The informal teaching conveyed by the house physician or house surgeon to his clerks or dressers during the morning round is a factor in clinical education almost equal in importance to the instruction given by the visiting staff at their state visits. The resident medical officers in a large teaching hospital are in close and continuous touch with the students under them. It is their special duty to impart elementary lessons in the grammar, as it were, of physical examination and diagnosis, leaving the finer points of syntax for their seniors to expound. They thus pave the way for the formal teaching of the physicians and surgeons; and the value of this largely depends on the thoroughness with which the residents perform their part. As pupil teachers in the great school of physic, they wield much power for good or for evil, not only by precept, but also by example.

Firstly, with regard to precept, and to the aims and limits of the house officer's teaching. The house physician cannot include the whole field of medicine in his instruction. He should confine himself to clinical pathology and the physical signs in chest, abdomen, and nervous system. It is outside his province to go deeply into morbid anatomy and differential diagnosis, and it is equally a mistake for him to compete with cram-books and epitomes. If, at the end of their first three months, his clerks can go over a case methodically, and can recognise such common physical signs as bronchial breathing, friction, a presystolic murmur, optic neuritis, or an aortic pulse, and can stain ordinary bacteria and do a blood-count or a Widal's test with accuracy, he has done his duty by them so far as actual instruction goes.

Similarly the house surgeon need not try to impart much book knowledge to his dressers. He should first drill them thoroughly in the principles and practice of asepsis. Then he should teach them to describe in precise language the injuries, morbid conditions, and operations that they have seen, and to recognise and apply the commoner instruments and appliances. He may leave the rest of surgery to be read in books or learnt by experience or taught by his chief.

With regard to example, the house officer who sets a high standard of professional conduct, by careful and systematic investigation, by the exercise of a critical and deliberate judgment, and by punctuality and kindness, exerts a lasting influence for good upon those students fortunate enough to hold their first clinical appointment under him. On the other hand, slovenly examinations, haphazard and premature diagnoses, dilatoriness and want of consideration for others, have a most pernicious effect upon the receptive minds of younger men. It is the first step that counts, in clinical medicine as in all else. Habits, good or bad, acquired by imitation in the early days of hospital work, soon become fixed beyond recall. The house surgeon who keeps in mind this responsibility towards his dressers, and lays himself out to set a good example to them, usually finds at the end of his term of office that his own powers of observation and deduction and his general clinical acumen have developed beyond all expectation.

In most of the great teaching hospitals the personal relations between the residents and the students are of the friendliest. In the wards discipline is maintained, and the house surgeon's wishes are regarded as commands, to be obeyed without question. This is an unwritten law, on the observance of which the work of the whole hospital depends; and it is respected with the utmost loyalty and good humour. Outside the wards the resident officer assumes no airs of superiority or authority, and is treated by his clerks and dressers as one of themselves. Often they are old school friends or college acquaintances, whom he has outstripped in the race for qualification. Often, too, the intimate relationship between house surgeon and dresser is the beginning of a life-long friendship.

Thus in these hospitals—and they form the large majority—a definite but unexpressed arrangement of give and take exists between the junior staff and students. The residents instruct the students to the best of their ability in the rudiments of their art, and afford them every opportunity for seeing and doing things for themselves. In return for this the latter co-operate loyally with their superior officers, relieving them of many tedious small duties, faithfully carrying out their wishes, and reserving all chaff and criticism, however well deserved, for use outside the wards.

But there are other hospitals, less rich, perhaps, in tradition, less fortunate in tone, in which from time to time the students and residents are not on such friendly terms. Here the students only obey those orders which suit their own convenience, while the residents maintain at all times an intolerable attitude of superiority, quite out of proportion to their merits or their position. The continuance of this absurd state of affairs is probably due to faults on both sides. The origin of the trouble may have been some thoughtless comment, overheard, passed on, distorted beyond recognition, and finally interpreted into a declaration of war by the leaders of the injured party. Whoever was in the wrong, it behoves the residents, as being the senior men and responsible for the good name of the hospital, to make the first overtures of peace.

